# Awake Proning: A Necessary Evil During the COVID-19 Pandemic

**DOI:** 10.7759/cureus.8989

**Published:** 2020-07-03

**Authors:** Sheharyar Khan, Erum Choudry, Syed Uzair Mahmood, Aisha Y Mulla, Syeda Mehwish

**Affiliations:** 1 Family Medicine, Baqai Medical University, Karachi, PAK; 2 Dentistry, The Indus Hospital, Indus Hospital Research Center, Karachi, PAK; 3 Pediatrics, The Indus Hospital, Indus Hospital Research Center, Karachi, PAK; 4 Dentistry, Karachi Medical and Dental College, Karachi, PAK

**Keywords:** mechanical vent, ards, hypoxia, coronavirus, lung injury

## Abstract

The spread of COVID-19 has been exponential throughout the globe. Though only a small percentage of infected individuals reach the critical stage of the disease, i.e., acute respiratory distress syndrome (ARDS), this percentage represents a significant number of patients that can overwhelm the healthcare system. Patients presenting with ARDS need mechanical ventilation, as their lungs are unable to oxygenate blood on their own due to fluid accumulation. One way to manage this excess pressure of fluid build-up around the lung tissues is to relieve the dorsal alveoli by prompting the patient to lie face down on the stomach; this is called awake proning. It is a procedure that is directed towards the recruitment of lung parenchyma when infected with pneumonia or when the condition has worsened into ARDS. This helps in relieving the pressure from the dorsal lung surface that has markedly higher perfusion than the ventral surface. Awake proning delays the use of mechanical ventilation and facilitates the patients with severe ARDS or severe pneumonia in maintaining the supply of oxygen to the body tissues. Since medical institutes are overburdened and limited ventilators are available, awake proning can reduce not only the burden on hospitals but also decrease the need for ventilators.

## Introduction and background

COVID-19 is a respiratory viral illness that is responsible for taking the lives of more than 8,064,000 people all around the world as of June 15, 2020. Becoming a rampant worldwide phenomenon, the number of patients with COVID-19 rises exponentially on a daily basis [1]. It is caused by a novel coronavirus named severe acute respiratory syndrome coronavirus-2 (SARS-CoV-2) [2]. The illness that it causes starts with mild symptoms like fever, dry cough, and sore throat and, in several people, with a recent loss in the senses of smell (anosmia) and taste (ageusia) [3-4]. As it progresses, the disease presents with more severe symptoms like viral pneumonia, which causes acute respiratory distress syndrome (ARDS).

ARDS is a condition of distress in respiration that results in poor ventilation, as well as poor perfusion of oxygen into the alveoli and, therefore, into the bloodstream. This poor perfusion causes hypoxic conditions, and congestive chest pain occurs along with a sense of drowning. The lungs are filled with exudate due to pneumonia and the alveoli start collapsing, which makes the condition even worse. Once a patient presents with ARDS and needs mechanical ventilation via intubation, the survival rate drops from 96% to 33% [5]. To prevent hypoxia and continue the process of gaseous exchange, it is vital that the effect of the exudate filling the lung parenchyma is decreased, and awake proning plays a major role in rescuing the lungs.

Awake proning limits the lung collapse by making the dorsal alveoli available for gaseous exchange. The patient is made to lie on their stomach, which has been proven to be of value in decreasing the rate of intubation according to several studies [6].

## Review

Pathophysiology of ARDS

Acute respiratory distress syndrome is a condition that results in pulmonary deficiency and in the case of severe symptoms, a need for mechanical ventilation arises. It is an acute and progressive onset of hypoxemia that is detected by the presence of bilateral infiltrates on the chest X-ray or a computed tomography (CT) scan. ARDS presents with a marked increase in the vascular permeability of the capillaries in the alveoli. This increased permeability is due to the damage caused to the endothelial layers of the capillaries, which result in an increased fluid passage into the alveolar lumen. Apart from vascular tissue damage, the excessive release of factors like sphingosine-1 phosphate (s1P) that binds to its receptor, S1P1, which regulates vascular permeability. Regarding the basic physiology of vascular stability, angiopoietin-1 (Ang-1) attaches to its receptor, tie-2, and stabilizes the vascular structure as the blood flows through the capillary bed by the activation of Syx and Rho A. Angiopoietin-2 (Ang-2) competes with Ang-1 to bind with the receptor sites at tie-2 and promotes the destabilization of the vascular structure. Hence, factors like s1P and Ang-2 are indicators of ARDS [7].

The body’s natural inflammatory response and the cells responsible for innate immunity like neutrophils are also responsible for the condition to escalate into ARDS. Upon activation, neutrophils release the molecules that are cytotoxic in nature, like enzymes, bioactive lipids, cytokines, and reactive oxygen species. These molecules, when released in excess, are responsible for cell necrosis and tissue damage. These damaged tissues induce autophagy as well as apoptosis, which are classical markers of ARDS [8]. Table 1 lists the biomarkers of ARDS that can be identified in serum [9].

**Table 1:** Biomarkers of ARDS ARDS: acute respiratory distress syndrome

Biomarkers of ARDS
1. Sphingosine-1-phosphate (S1P)
2. Rho GTPases (RhoA/ROCKs)
3. Proinflammatory cytokines such as TNFα, IL-1β, and IL-8
4. Angiopoietin-2
5. Angiotensin-converting enzyme 2 (ACE II)
6. Bioactive lipids
7. Receptor for advanced glycation end-products (RAGE)
8. Surfactant protein D (SP-D)

All the instances mentioned above result in the rapid increase of fluid accumulation inside the thoracic space surrounding the lung tissue, and the fluid presses against the alveoli. The shape of the lungs inside the human body favors heavy perfusion towards the back or dorsal side of the body. There is a comparatively lower rate of perfusion towards the front or ventral side of the body. When a patient is diagnosed with pneumonia or ARDS, the fluid collected in the thoracic space puts additional pressure on the delicate alveoli, which may lead to alveolar collapse [10].

In addition to an increase in pressure over alveoli, fluid also hinders efficient gaseous exchange to meet the requirement of the body. Together, these factors cause a decreased availability of oxygen for the tissues and results in hypoxemia. Hence, the patients diagnosed with pneumonia or ARDS often present with atelectasis (lung collapse) in the dorsal region. This condition arises because the patient, lying supine, accumulates fluid in the dorsal alveoli, i.e., the region with higher perfusion [11].

How does awake proning work?

In ARDS, there is an excessive accumulation of secretion in the peripheral lung parenchyma, which exerts pressure against the fragile alveolar walls and impedes gaseous exchange. A method to redirect this excessive fluid away from the dorsal lung parenchyma is to manage it via awake proning, which is majorly directed towards conserving the alveolar structure of the lungs [10]. The procedure is focused on limiting the collapse of the alveoli and reducing the fluid accumulation on the areas with a higher perfusion rate, i.e., the dorsal region. The prone position is achieved when the patient lies on their stomach or front, this helps in the recruitment of the alveoli previously collapsed into the dorsal surface of the chest cavity [12].

**Figure 1:**
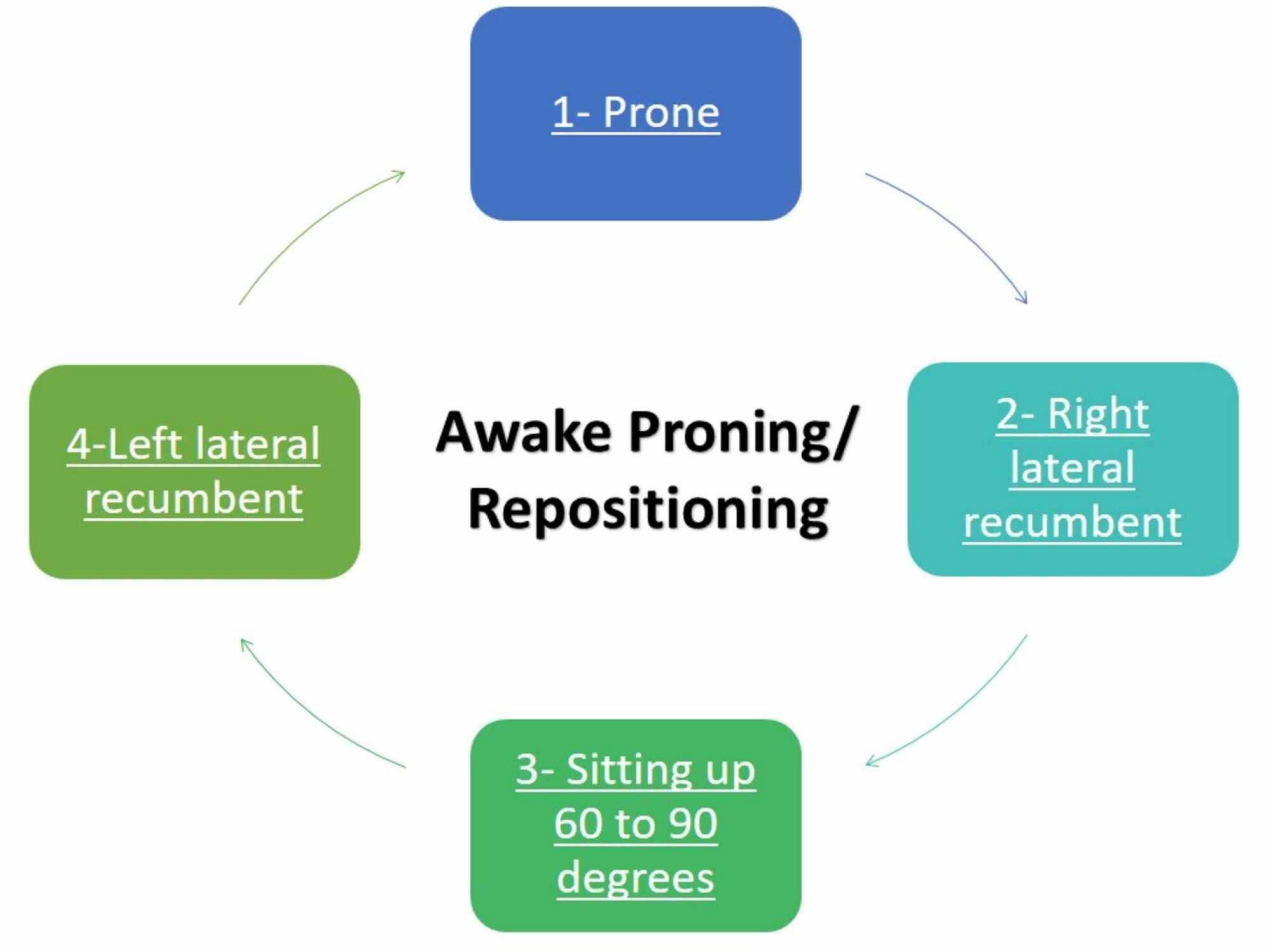
Steps of awake proning Note: Reassess the respiratory rate and saturation after 10 minutes of repositioning the patient and later after every 10 to 20 minutes.

In prone positioning, the intra-abdominal organs move under gravitational pull in a downward (gravitational) and forward (toward the thorax) direction, which aims the excess pleural pressure at the ventral region of the alveoli as opposed to the dorsal region in the case of lying in a supine position. Redistribution of the pressure towards the ventral region also helps in preventing the compression of dorsal alveoli and aids in better breathing [10]. Figure 1 illustrates the steps of awake proning.

Benefits of awake proning

Awake proning is associated with an improved mortality rate after an incident of ARDS or severe pneumonia. This procedure is noninvasive and provides instant results. In emergency situations where the patient's vitals keep worsening, applying prone positioning helps in improving the oxygen saturation instantly. Several studies show that awake proning improves oxygen saturation in merely five minutes [13].

1. It maintains an optimal respiratory rate and enhances the gaseous exchange in a favorable range [14].

2. Ventilation stays homogenous throughout the lung parenchyma, and redistribution of the blood flow is improved with higher efficacy, which, in turn, improves the ventilation/perfusion ratio (V/Q ratio).

3. An intrapulmonary shunt prevents the proper oxygenation of blood in the lungs. The areas shunted are prone to hypoxia and may result in tissue damage. By prone positioning, this shunt is reduced and lung compression is decreased. Therefore, oxygen levels improve [15].

4. Awake proning also helps decrease the accumulation of excess interstitial fluid in the dorsal part of the lungs. Lying on the stomach directs the fluid to collect in the ventral region where there is comparatively less perfusion [16].

5. No specialized instruments are needed for the procedure, and it can be done easily in emergency situations [16].

Evidence of lowered mortality rate due to awake proning

A recent study conducted in New York regarding the effectiveness of self-proning came out with promising results. The study was based on quick proning of patients that presented with moderate to severe ARDS after an incident of COVID-19. The patients were put into the prone positions for about 18 hours a day while the breathing cycles started showing improvements after five minutes of assuming the position of awake proning. Fifty patients with confirmed hypoxia were under focus for this study and their median saturation of oxygen in blood was 80%, which was raised to 84% after the provision of supplemental oxygen. After five minutes of lying in the prone position, the levels of oxygen saturation were raised to 94%, and the patients were also put on supplemental oxygen. In this study, intubation was not needed in nearly two-thirds of the admitted patients. These patients were shifted towards the non-invasive procedures of oxygen provision like bi-level positive airway pressure (BiPAP) and awake proning [13].

Another study focused on 15 patients who were not intubated. These patients were subjected to prone positioning 43 times over the course of treatment. The patients presented with ARDS and breathing difficulties. The respiratory rate and blood oxygenation were substantially improved, during and after the cycle of pronation and there were patients who had improved breathing after pronation. The blood oxygen levels also improved after the cycles of prone positioning, and endotracheal intubation was avoided in patients with ARDS, which would have been the only option to opt from if awake proning was not administered [17].

Furthermore, a study had 50 subjects with COVID-19 and severe ARDS. The median blood oxygen levels were found to be 80% and after the aid of supplemental oxygen, these levels improved to 85%. Prone position was maintained, and the blood oxygen before and after proning was noted. At five minutes of proning, the levels went up to 94%. In this study, however, 13 patients failed to respond to the treatment by proning and had to undergo endotracheal intubation. For emergency support, the numbers suggest that proning provided improved levels of oxygen saturation in the patients who would have been treated with endotracheal intubation and mechanical ventilation [18].

Indications of awake proning

One of the indications of awake proning is the need for quick relief from the dyspneic condition. COVID-19 patients in a critical condition escalate into moderate, severe, or critically severe ARDS. This calls for the need for a quick fix until their condition stabilizes [19].

The two types of awake proning are indicated for different situations:

A. Short-term awake proning:

It may have limited use but there are instances when short-term awake proning is the best option to handle the patient’s condition. The time period of short proning spans from three hours to eight hours [17,20].

1. It is indicated to treat mild to moderate hypoxemia.

2. It helps in airway drainage and improves refractory maneuvers in relation to atelectasis.

3. Lower lobe atelectasis is most effectively treated with short-term prone positioning.

4. Improved breathing rate and decreased crackles during each breathing cycle can be observed.

B. Long-term awake proning:

It is the most widely used maneuver to administer awake proning and has shown the most significant results. The time period of long-term awake proning spans for more than eight hours. 

1. It is indicated to treat severe hypoxemia.

2. Severe ARDS is most effectively treated with long-term awake proning. The condition is characterized by a steep decline in blood oxygenation and severe dyspnea indicating loss of efficient breathing. It is the last stage in the development of COVID-19 infection [14].

Contraindications

Awake proning is contraindicated in the following situations [21]:

1. A history with spinal instability like spondylolisthesis, scoliosis, injury, or trauma to the spine

2. Increased intracranial pressure

3. Pregnancy

4. Hemodynamically unstable conditions like hypertension and cardiopulmonary diseases

5. Abdominal open wounds

However, the presence of these contraindications should be balanced with the need for the treatment. The risks associated with awake proning should be considered in relation to the necessity of the procedure at the time of treatment, making it a necessary evil. 

Complications associated with awake proning

Physicians may encounter a few complications associated with awake proning, as illustrated in Table 2 [22]:

**Table 2:** Complications associated with awake proning ECG: electrocardiogram

Table 2: Complications associated with awake proning
1. Decrease in the provision of enteral nutrition
2. Chest tube dislodgement
3. Increase in Intra-abdominal pressure
4. Monitoring difficulty e.g. ECG monitoring
5. Facial edema
6. Pressure trauma on the nasal bridge, mentum, humeral head, knees, and male genitals
7.Increased intracranial pressure (ICP) leading to blindness

Why not "sleep" proning

A setback of lying prone for an extended period is that it causes pain in the back, neck, and lower limbs. This pain is caused by the body’s weight being projected on to the spine. Sleeping in a prone position hinders the spine to position itself accurately and leads to a multitude of problems. If the spine remains unstable, the nerves exiting the spinal segment might impinge and cause pain in the area it supplies. This could feel like tingling or numbness and, in extreme cases, may cause severe pain. In rare cases, the airway can be obstructed and it may present as obstructive sleep apnea. It is recommended that patients suffering from ARDS be advised to acquire a prone position while they are conscious but while falling asleep, a neutral position should be advised [23].

## Conclusions

A COVID-19 patient presenting with severe pneumonia or ARDS can be managed with awake proning as a supportive treatment to relieve symptoms. Awake proning helps in improving oxygenation by the optimization of the lung parenchyma and the recruitment of the alveoli along the dorsal surface with higher perfusion, hence, better ventilation is provided to the body. Prone positioning helps in protection against ventilator-induced lung injury (VILI), as the need for mechanical ventilation is avoided for some time by the distribution of stress and strain in a homogenous manner throughout the parenchyma of the lungs. The long-term use of awake proning is, however, not indicated for mild and moderate cases of ARDS. Awake proning does not require any special instrumentation and can be done in an emergency situation. It presents itself as a tool that can improve the oxygen saturation of a patient suffering from hypoxemia due to conditions such as ARDS. With the shortage of ventilators and their high demand during the COVID-19 pandemic, we recommend that each medical institute should invest in or should seek help to acquire prone position comfort cushions or bolsters as they help maintain awake proning rather than opt for expensive ventilators.
